# Picobirnavirus: how do you find where it’s hiding?

**DOI:** 10.1080/1040841X.2025.2560918

**Published:** 2025-09-20

**Authors:** Abbey L. K. Hutton, Susanna Grigson, Louise Bartle, Bhavya Papudeshi, Vijini Mallawaarachchi, Anita Tarasenko, James G. Mitchell, Robert A. Edwards

**Affiliations:** a Flinders Accelerator for Microbiome Exploration, College of Science and Engineering, Flinders University, Bedford Park, Australia; b School of Agriculture, Food and Wine, The University of Adelaide, Urrbrae, Australia

**Keywords:** Picobirnavirus, PCR, metagenomic sequencing, RdRp, bacteriophages

## Abstract

Picobirnaviruses (PBVs) are double-stranded RNA viruses detected in various environments and host-associated samples, including those from humans, non-human animals, invertebrates and birds. First described in human fecal material, PBVs were initially hypothesized to be human enteric pathogens. However, no definitive association with disease has been established. Their pathogenic potential remains unclear, therefore, their presence in clinical or environmental samples may reflect asymptomatic colonization, indirect association or infection of a non-human host. The PBV genome exhibits remarkably high genetic diversity both within and across its genomic segments, as well as notable variability in genetic code usage. Some PBV genomes use alternative codon assignments, raising the possibility that they infect prokaryotic or otherwise unconventional hosts. This review critically examines the experimental and bioinformatic methods used to detect PBVs and infer their host range. We distinguish between methods used for PBV genome identification (e.g. PCR, metagenomic sequencing) and those aimed at host determination (e.g. culturing attempts, codon usage bias, cloning into model systems). We also evaluate the challenges and limitations associated with each approach. Elucidating PBVs’ host range is essential to understanding their biological roles and ecological significance, including potential implications for human and animal health and microbial community dynamics across ecosystems.

## Introduction

**Figure 1.**
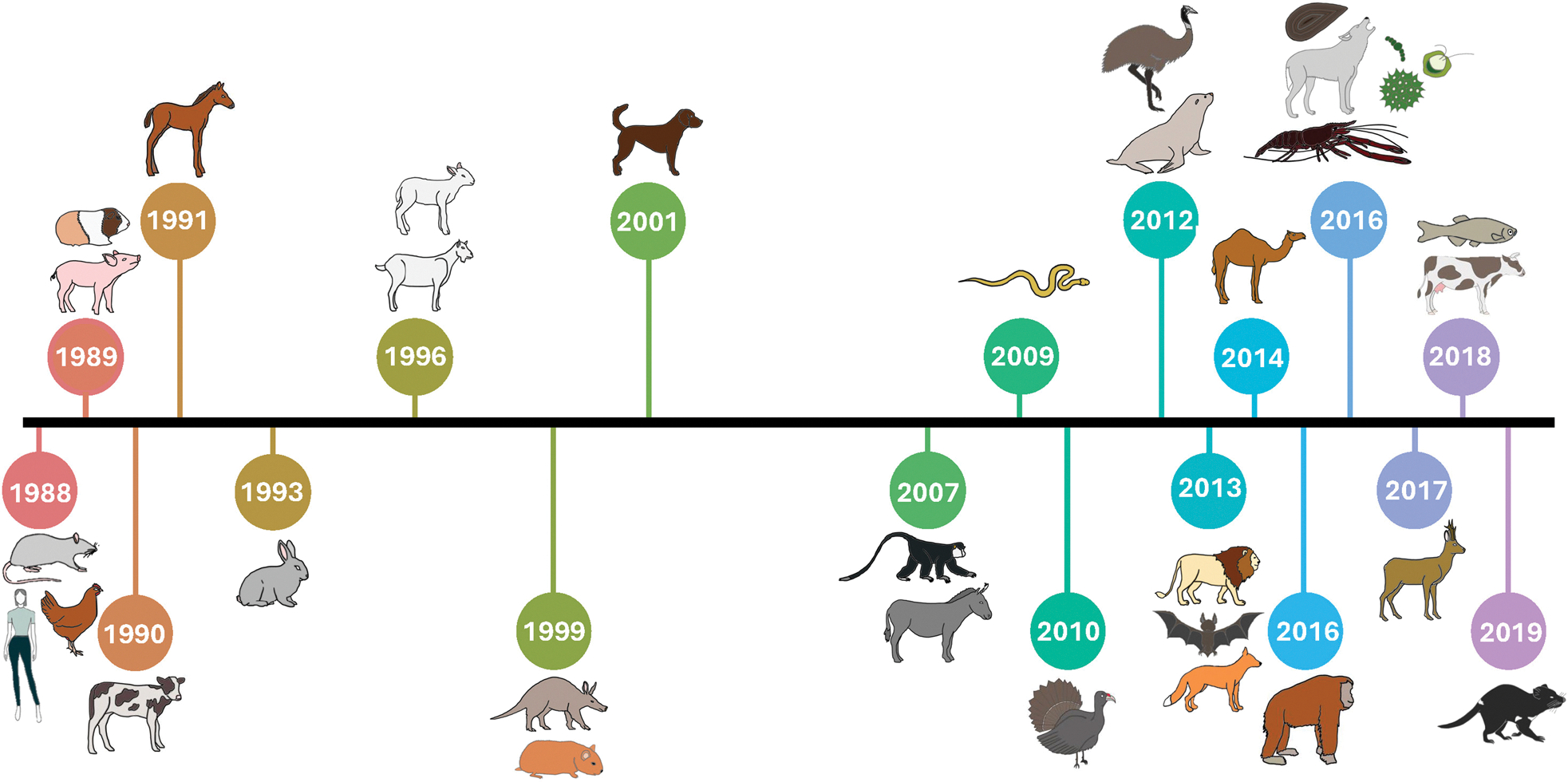
Timeline of PBV identification in different taxa. Animals not to scale. (Pereira et al. 1988; Gatti et al. 1989; Green et al. 1999; Haga et al. 1999; Volotäo et al. 2001; Masachessi et al. 2007; Fregolente and Gatti 2009; Day and Zsak 2014; Woo et al. 2012; Gillman et al. 2013; Masachessi et al. 2015; Navarro et al. 2018; Shi et al. 2016, 2018; Chong et al. 2019; Kashnikov et al. 2020).

*Picobirnavirus* (PBV) has traditionally been considered an enteric pathogen. Initially identified in human and animal fecal samples, it has been associated with diarrheal illness (Smits et al. 2012; Delmas et al. 2019). Since their discovery in 1988 *via* RNA PAGE, PBVs have been detected in many environments and hosts, including in invertebrates, birds, and wastewater, as well as in asymptomatic individuals (Pereira et al. 1988; Ganesh et al. 2014; Shi et al. 2016; Adriaenssens et al. 2018; Yinda et al. 2018; Delmas et al. 2019; Guajardo-Leiva et al. 2020). A timeline of key PBV dis-coveries across different host organisms is summarized in Figure 1. These findings have raised important questions about the true host range of PBVs and the true nature of their pathogenicity.

*Picobirnaviruses* (PBVs) are typically bi-segmented double-stranded RNA viruses. Segment 1 (2.2–2.7 kb) encodes the capsid protein and two additional, functionally uncharacterized proteins, while segment 2 (1.2–1.9 kb) encodes the RNA-dependent RNA polymerase (RdRp) (Smits et al. 2012; Delmas et al. 2019; Ullah et al. 2022). However, several unsegmented PBV-like genomes with fused segments have been identified in species such as robins (Ullah et al. 2022), horses (Li et al. 2015) and marmots (Luo et al. 2018), suggesting that PBV genome architecture may be more variable than previously recognized.

Despite being detected in individuals with gastrointestinal disease, PBVs have not been definitively linked to any specific disease. Nevertheless, some studies suggest PBVs may be associated with disease under certain conditions. For example, PBV was detected in 40% of individuals undergoing organ transplantation, and its presence was proposed as a strong predictor of severe enteric graft-versus-host disease (GvHD) (Legoff et al. 2017). Similarly, PBV sequences were detected in stool samples from HIV-positive patients with gastrointestinal symptoms (Giordano et al. 1998), although the detection rate (8.8%) may have underestimated the presence of PBV due to the sensitivity of sequencing methods available at the time (Giordano et al. 1998).

In animals, PBVs have been found in both symptomatic and asymptomatic individuals, particularly in captive populations, suggesting stress or immune suppression may influence viral excretion (Malik et al. 2014; Gisela Masachessi et al. 2015; Gallagher et al. 2017; Chagas et al. 2024; Qian et al. 2024; Reddy et al. 2025). These findings have led to speculation that PBVs may act as opportunistic pathogens in immunocom-promised hosts, although their precise role remains unsolved. In addition to enteric samples, PBVs have also been detected in the respiratory tract of pigs and humans (Smits et al. 2012, 2011), challenging the notion that they are strictly gastrointestinal viruses.

Given the absence of a known host, it remains unclear whether PBVs infect multicellular eukaryotes, microbial hosts such as bacteria or fungi, or behave as commensal or opportunistic agents under certain conditions. Identifying the host(s) of PBVs is crucial to understanding their biology, potential pathogenicity, transmission dynamics and ecological significance. In this review, we use the term “*picobirnaviruses*” (PBVs) to refer to the viral family as a whole; “*picobirnavirus*” refers to an individual virus within this group; and “PBV” is used as a general abbreviation for the family or referring to the group collectively.

This review critically evaluates the evidence for PBV host identification, focusing on genome characteristics such as codon usage, segment diversity and potential implications of nonstandard genetic codes. We assess the current methodologies used to detect and characterize PBVs – including direct culturing, PCR, metagenomic sequencing, and cloning into model systems – and discuss their strengths, limitations, and implications for resolving the enigmatic host-virus relationship.

## Genomic diversity

Although initially proposed to infect animal cells, no PBVs have been successfully cultured to date (Krishnamurthy and Wang 2018). As a result, much of our current understanding of their biology is derived from genome analysis.

Originally, PBVs were categorized into two genogroups (GI and GII) based primarily on sequence data (Malik et al. 2014; Boros et al. 2018). However, more recent whole-genome analyses – including both genome segments – have suggested the existence of a third genogroup (GIII) (Ullah et al. 2022; Perez et al. 2021). These three genogroups are defined phylogenetically, based on sequence divergence and clustering of viral genomes (Malik et al. 2014; Boros et al. 2018; Ghosh and Malik 2020; Ullah et al. 2022). PBV GI and GII sequences are most commonly derived from vertebrate-associated samples, while GIII sequences are typically found in invertebrates (Perez et al. 2021; Ullah et al. 2022).

Other studies have proposed the existence of up to five genogroups, based on maximum-likelihood phylogenetic trees of full RdRp sequences that consistently form five distinct clades (Li et al. 2015; Luo et al. 2018). Moreover, PBV-like sequences utilizing alternative mitochondrial genetic codes have been identified, placing them outside the conventional genogroups and raising the possibility of non-vertebrate hosts such as fungi (Krishnamurthy and Wang 2018; Yinda et al. 2018; Ullah et al. 2022; Reddy et al. 2025).

Taken together, these findings demonstrate that the precise number and composition of PBV genogroups remains unsolved and may depend on methodological differences, sampling biases, or the ongoing discovery of novel lineages. Further studies using standardized phylogenomic approaches and expanded sampling will be essential to clarify the true diversity and evolutionary relationships within this viral group.

## Phylogenetic classification

While many studies have attempted to resolve the phylogenetic context for *Picobirnaviruses*, recent large-scale RNA virus analyses have provided a clearer view of their placement within the broader virosphere. Hou et al. (2024) developed LucaProt, an AI-based tool that integrates both RdRp protein sequences and predicted structural features to detect highly divergent RdRp sequences from global metatranscriptomic datasets. This study identified a viral supergroup containing both Partitiviruses and Picobirnaviruses (Hou et al. 2024). However, within this supergroup, PBVs form a distinct clade, with Partitiviruses positioned separately, suggesting they act as a phylogenetic outgroup. This finding aligns with earlier work by Wolf et al. (2018), which also placed PBVs within a broader “picornavirus supergroup” encompassing both *picobirnaviruses* and *partitiviruses.* Together, these studies highlight the consistency of this relationship across independent, large-scale RNA virus phylogenetic frameworks, supporting the idea that PBVs may have evolved from a partitivirus-like ancestor or common origin, rather than direct lineage. Adding further to this interpretation, Sadiq et al. (2024) identified multiple PBV genotypes co-occurring with partitivirus-like sequences in wastewater viromes, suggesting the possibility of ecological overlap and niche similarity.

**Figure 2.**
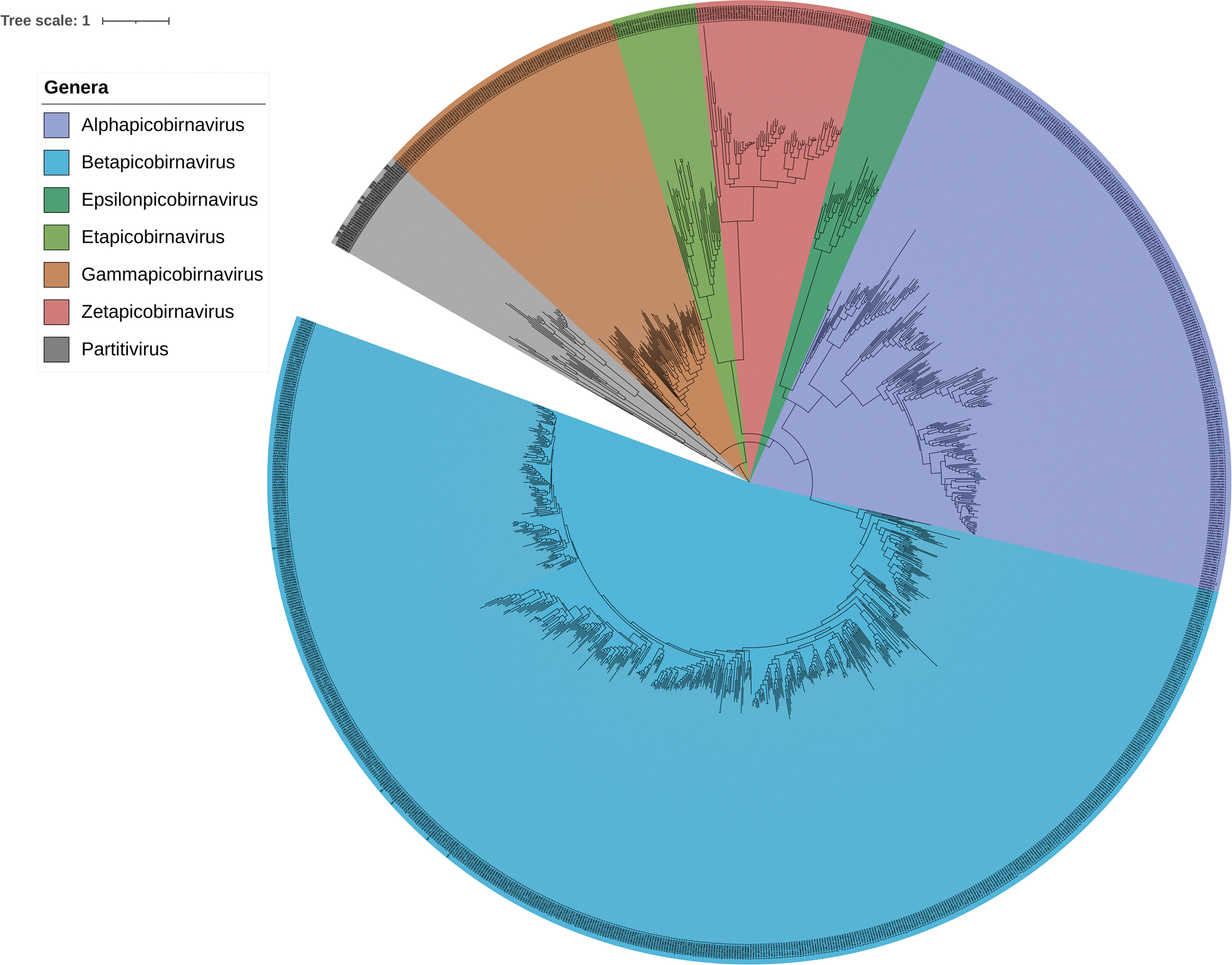
Maximum likelihood phylogenetic tree of PBV and *partitivirus R*dRp protein sequences from the NCBI database previously described by Sadiq et al. (2024). Sequences were aligned using MAFFT, the tree was constructed using FastTree (JTT model, SH-like local support values) and visualized using iTOL v6.

To investigate the relationship between picobirnaviruses and partitiviruses, a dataset comprising 1103 RdRp protein sequences was retrieved from GenBank using NCBI (Brister et al. 2015), based on sequences reported by Sadiq et al. (2024). The dataset included approximately 50 partitiviruses and 1,050 picobirnavirus sequences. A multiple sequence alignment was performed using MAFFT with default parameters (Katoh and Standley 2013), and a maximum likelihood phylogenetic tree was constructed using FastTree version 2.1.11 with the JTT model of amino acid substitution and a CAT approximation with 20 rate categories (Price et al. 2010). The resulting tree was visualized using Interactive Tree of Life (iTOL) v6 platform (Letunic and Bork 2021). The tree (Figure 2) delineates the separation of picobirnaviruses into distinct clades, with label coloring based on the newly proposed classification groups described by Sadiq et al. (2024). This analysis demonstrates that PBV sequences cluster into six distinct clades, in contrast to the three traditional genogroups (GI-GIII) previously described. The partitiviruses form a separate, well-supported clade, further supporting the previous findings that, although PBVs and partitiviruses share a common ancestry, they represent distinct evolutionary lineages.

## Host hypotheses

Determining the host range of PBVs remains a fundamental challenge in virology. One of the key obstacles is the use of nonstandard genetic codes in some PBV genomes, which complicates both genome annotation and host inference (Kleymann et al. 2020; Perez et al. 2021; Ullah et al. 2022; Krishnamurthy and Wang 2018; Boros et al. 2018; Yinda et al. 2018; Adriaenssens et al. 2018). While some PBVs utilize the standard eukaryotic genetic code (NCBI translation Table 1), others appear to adopt alternative codon tables, raising questions about whether PBVs infect bacteria, archaea or eukaryotic organisms.

One hypothesis suggests that PBV infect fungal mitochondria, akin to *mitoviruses* – RNA viruses known to replicate within fungal mitochondrial compartments. For instance, Ullah et al. (2022) reported a unique, unsegmented PBV from the wild robin *Luscinia obscura*, that appeared to use the yeast mitochondrial genetic code (translation Table 3), implying a fungal mitochondrial host. *Mitoviruses* replicate exclusively within the mitochondria and are typically transmitted vertically, with limited horizontal transfer (Yinda et al. 2018; Reddy et al. 2023). The resemblance between PBV and *mitovirus* replication cycles is further supported by codon usage bias analysis, where PBV RdRp genes clustered with those of *mitoviruses* in principal component analyses comparing codon Tables 3, 4, 5, 9, and 13 (Yinda et al. 2018). Supporting this, some PBVs (e.g. those found in bats) lack identifiable capsid-encoding open reading frames (ORFs), a feature consistent with the non-encapsulated nature of *mitoviruses* (Yinda et al. 2018). However, a majority of PBV genomes do encode a capsid and display icosahedral virion structure, suggesting that the absence of a capsid in some genomes may reflect annotation challenges or natural genomic variation, rather than an absence of extracellular virions.

An alternative hypothesis proposes that PBVs may infect prokaryotic hosts. This view is supported by growing evidence of codon reassignment in bacteriophages (Ivanova et al. 2014; Devoto et al. 2019; Yutin et al. 2021; Borges et al. 2022; Cook et al. 2024). For example, several phage families exhibit reassigned codons in structural and lysis genes, potentially serving as regulatory elements during lysogenic-lytic transitions (Borges et al. 2022). PBVs may exhibit similar genome-wide codon reassignment patterns, which would be consistent with a prokaryotic mode of infection.

Additional support for a bacterial host comes from the identification of ribosomal binding site (RBS) motifs – particularly Shine-Dalgarno sequences – in PBV genomes (8). Krishnamurthy and Wang’s (2018) demonstrated that PBVs harbor a high frequency of the RBS motifs, a hallmark of bacterial gene regulation. Indeed, the *Picobirnaviridae* family exhibited greater enrichment of RBS motifs than many confirmed prokaryotic families, suggesting a potential adaptation to bacterial translation mechanisms (5, 13, 44). One study found that between 83 and 85% of predicted ORFs contained RBS motifs, a level of conservation consistent with bacterial hosts such as *Proteobacteria* or *Firmicutes*, where 75–85% of genes are preceded by canonical RBS elements (Krishnamurthy and Wang 2018). The prevalence and conservation of these motifs imply strong evolutionary selection pressures from a bacterial genomic context.

**Figure 3.**
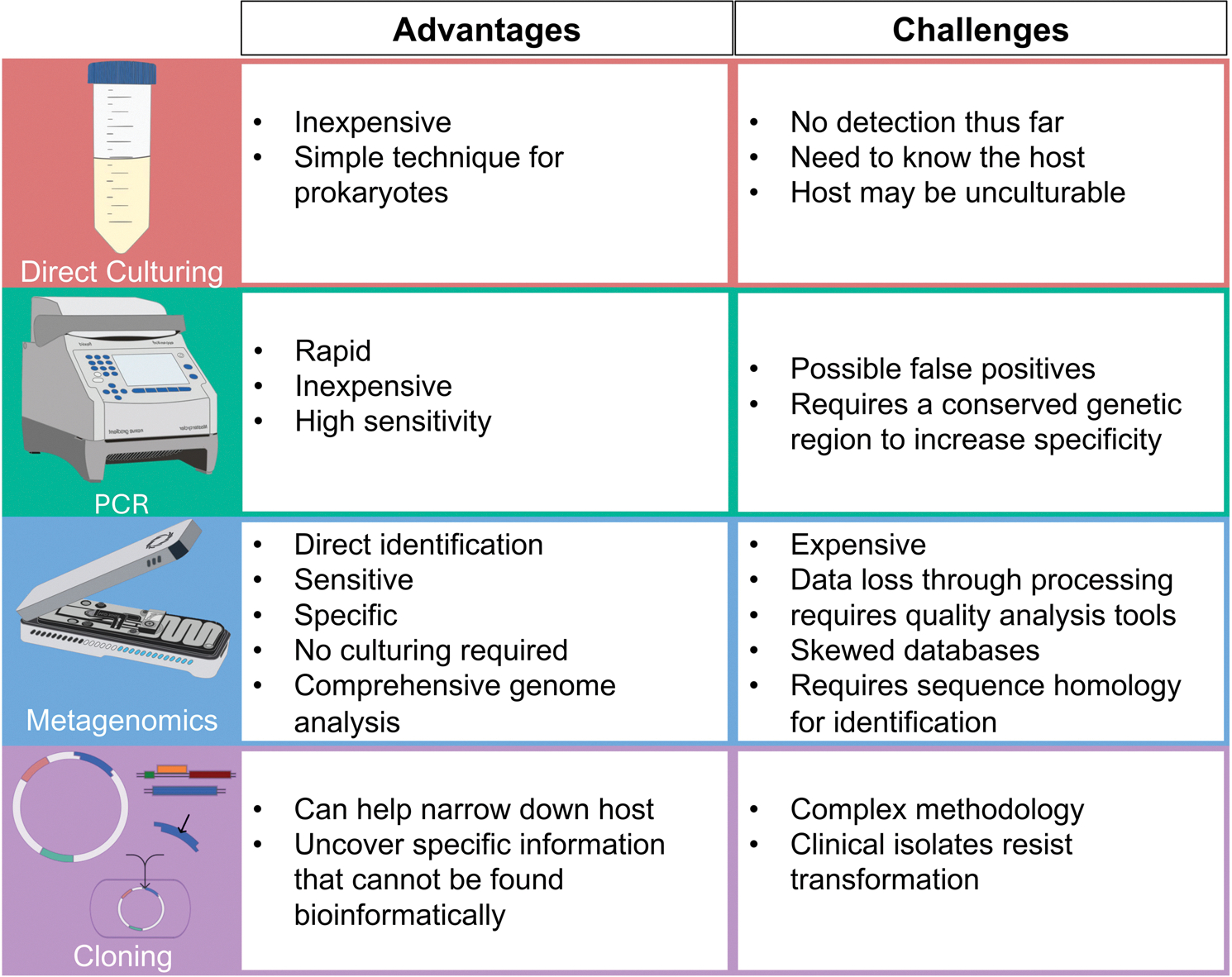
Advantages and challenges of the current experimental methods used in the identification of *picobirnavirus.*

While the possibility of archaeal hosts has not been ruled out, current data strongly support either fungal mitochondrial or bacterial hosts. Disentangling these possibilities will require additional experimental data, which could include *in situ* localization, host-virus association *via* CRISPR spacer matches, and single-cell metagenomic studies to co-localize PBV genomes with potential matches. However, as these methods have yet to be utilized to address this question, researchers have relied on a variety of other detection methods to try to characterize PBVs. A summary of the current experimental and bioinformatic approaches used for the detection and characterization of picobirnaviruses, including their advantages and challenges, is provided in Figure 3.

## Detection methods

### Direct culturing

Directly culturing PBVs has been attempted on multiple occasions, but no successful replication has been reported to date. For example, Boros et al. (2018) attempted to culture PBVs from chicken cloacal samples by inoculating a 3% suspension into brain heart infusion medium. However, viral replication could not be detected *via* RT-qPCR (Boros et al. 2018).

Several factors may have contributed to the lack of detectable viral growth. The samples had been stored at − 20 °C prior to culturing, which may have resulted in RNA degradation or reduced viability of infectious particles. Moreover, the low concentration of intact, cultivable host cells and unknown requirements for optimal viral replication conditions further complicate cultivation efforts. Additionally, the inherently low viral load of PBVs in environmental or clinical samples may fall below the threshold required for successful propagation. These challenges underscore the limitations of direct culturing for PBVs and highlight the need for alternative methods to elucidate their biology and host range.

### PCR-based detection

Reverse transcription PCR (RT-PCR) and quantitative RT-PCR (RT-qPCR) have been central to PBV detection and quantification since the virus was first discovered (Pereira et al. 1988). These techniques are highly sensitive and offer rapid identification, with PCR products often used for downstream sequencing to characterize specific genomic regions (Payne 2017). Most assays target the conserved regions of the RdRp gene, which remains the primary molecular marker for PBV (Bányai et al. 2003; Carruyo et al. 2008; Ganesh et al. 2010; Martínez et al. 2010; Woo et al. 2016; Boros et al. 2018).

For example, Woo et al. (2016) designed primers targeting a 205 bp fragment of the RdRp gene and successfully detected them in a broad range of animal species, including cattle, monkeys, horses, pigs, rabbits, a dog and California sea lions. PBVs have also been detected in porcine fecal samples using RT-PCR, with many sequences showing Shine-Dalgarno motifs indicative of potential prokaryotic translation regulation (Carruyo et al. 2008; Martínez et al. 2010).

Despite its advantages, PCR has several limitations. The high genetic diversity of PBVs leaves only small, conserved regions suitable for primer design, restricting the scope of detection. For example, Reddy et al. (2025) screened 150 fecal samples from six animal species using multiple different primer sets targeting the RdRp gene. While RNA-PAGE detected PBV in buffalo and dog samples, RT-PCR revealed the highest prevalence in the goat samples (24%), despite the same samples testing negative by RNA-PAGE. This underscores the influence of primer selection on detection outcomes. Additionally, PCR’s high sensitivity makes it prone to contamination, potentially leading to false-positive results (Garibyan and Avashia 2013; Payne 2017). Furthermore, PCR is primarily a presence/absence assay and does not provide direct insight into viral host identity or pathogenicity. More advanced applications, such as *in situ* PCR or single-cell approaches, could potentially localize PBV genomes within host cells, providing direct evidence of host identity. However, such methods have not currently been used for PBVs, likely due to their low abundance, genomic diversity, and technical challenges in tissue-specific sampling. Despite these constraints, PCR remains a valuable tool in the detection of PBVs. It complements more comprehensive techniques, such as metagenomics next-generation sequencing, by enabling targeted amplification, supporting primer design, and aiding in the phylogenetic analysis of PBV sequences.

### Metagenomics and bioinformatics analysis

Given the challenges associated with culturing many viruses, metagenomic sequencing has become the primary tool for exploring viral diversity in unculturable taxa such as PBVs (Bassi et al. 2022; Roux et al. 2021; Wang D 2022; Mallawaarachchi et al. 2023; Roach et al. 2024; Dutilh et al. 2014; Grigson et al. 2023; Pargin et al. 2023). Through next-generation sequencing (NGS) researchers have directly recovered viral RNA from environmental or clinical samples and reconstructed genomes *via* computational pipelines (Bányai et al. 2003; Carruyo et al. 2008; Ganesh et al. 2010; Martínez et al. 2010; Woo et al. 2016; Boros et al. 2018; Knox et al. 2018; Xiao et al. 2020; Berg et al. 2021; Huaman et al. 2021; Ramesh et al. 2021; Chauhan et al. 2022; Knox et al. 2023; Sadiq et al. 2024). For instance, Ramesh et al. (2021) used metagenomics sequencing of porcine fecal slurries and found PBVs to be the most abundant viral genus, with <75% similarity between many recovered RdRp fragments. Similarly, Chong et al. (2019) identified six novel PBV genomes of Tasmanian devil feces, revealing high diversity and widespread phylogenetic dispersion of RdRp sequences.

However, metagenomics is constrained by low RNA abundance, sample degradation, and technical biases. Viruses typically constitute <5% of environmental sequence data (Forbes et al. 2017; Glickman et al. 2021), and PBV RNA often represents a minute fraction relative to host-derived nucleic acids such as DNA (Wang Y et al. 2021; Greninger 2018). Amplification and extraction steps can introduce bias or cause viral lysis, reducing recovery efficiency (Bassi et al. 2022). As a result, PBVs may be underrepresented in metagenomic datasets relative to their actual prevalence in biological samples, particularly when using outdated sequencing platforms or inadequate sample preparation (Hillary et al. 2022).

### Bioinformatic approaches and challenges

Following sequencing, bioinformatics tools are used to classify, annotate and infer taxonomy and function. Analyses of sequence homology, codon usage, and structural prediction have helped classify PBV strains and place them within the broader context of dsRNA virus evolution (Bányai et al. 2003; Carruyo et al. 2008; Ganesh et al. 2010; Martínez et al. 2010; Woo et al. 2016; Boros et al. 2018; Knox et al. 2018; Xiao et al. 2020; Berg et al. 2021; Huaman et al. 2021; Ramesh et al. 2021; Chauhan et al. 2022; Knox et al. 2023; Sadiq et al. 2024).

Some studies have investigated potential host interactions (Knox et al. 2018; Knox et al. 2023). Xiao et al. (2020) assembled both PBV genome segments from rabbit fecal samples, but were unable to generate a reliable phylogeny due to extreme sequence divergence in the capsid encoding region. This illustrates that even among confirmed PBVs, some genomic regions may share only weak homology with known sequences, making them difficult to detect or classify using standard homology-based tools. As such, PBVs with highly divergent capsid genes may be overlooked in metagenomic datasets, contributing to the underestimation of their diversity.

A study on Ugandan gorillas and cattle applied protein alignments and maximum-likelihood phylogenetic analysis to assess possible cross-species transmission events, but no conclusive evidence was found (Knox et al. 2018). Other studies have integrated host removal pipelines and Bayesian inference to explore clinical associations. Berg et al. (2021) identified a novel PBV strain in patients with and without respiratory infection, and linked specific RdRp genotypes to clinical presentations using qPCR and statistical association analysis (Berg et al. 2021). Despite these correlations, no causal relationship could be established, and PBVs were proposed as potential opportunistic or incidental agents.

In contrast, Neri et al. (2022) developed a computational pipeline to identify PBVs encoding bacteriolytic proteins – a hallmark of phages infecting bacteria. By using RdRp contigs as bait, they identified PBVs with lysis domains, including lytic transglycosylases or metallopeptidases (Neri et al. 2022). These findings lend support to the hypothesis that PBVs may be prokaryotic viruses.

### Limitations of current bioinformatic tools

PBVs present a unique challenge for current bioinformatic tools. Taxonomic classification relies on sequence similarity to reference databases, which are heavily biased toward well-characterised DNA viruses. Highly divergent RNA viruses like PBVs, especially those using alternative genetic codes, may not align with known genomes and therefore remain unclassified (Forbes et al. 2017; Smith et al. 2022).

Annotation tools that fail to accommodate genetic code variation can truncate genes, misclassify ORFs, and inflate hypothetical protein predictions. This is particularly problematic for segmented viruses like PBVs, for which the correct annotation of each segment is crucial. Using tools such as *Pyrodigal gv,* which allows for genetic code flexibility, may improve gene prediction accuracy and downstream analyses (Cook et al. 2024). Finally, identifying the host of PBVs remains particularly difficult *via* metagenomics alone. It is challenging to move beyond correlation without a physical linkage between the host and the viruses. The high diversity and segmentation of PBV genomes only amplify this issue.

## Cloning and functional characterization of PBV genes

### Cloning in bacteria

To overcome the limitations of direct detection and functional analysis, segments of the PBV genome have been cloned into bacterial systems. These efforts have aimed to both increase sequence yield for genomic analysis and explore protein expression potential.

For example, Boros et al. (2018) cloned several PBV ORFs into plasmids and expressed them in *Escherichia coli* (*E. coli*) to assess their transcriptional capacity. Western blot analysis using His-tag detection confirmed the expression of all six ORFs, although no infectious particles were produced, suggesting that while the viral proteins are expressible in bacterial systems, additional host factors are likely needed for full replication (Boros et al. 2018).

Leeuwen et al. (2010) also used bacterial cloning to amplify PBV genome fragments ranging in size between 200 and 1,500 bp. Cloning into *E. coli* facilitated the sequencing of both genome segments – a notable achievement given the difficulty of detecting both strands in many previous studies (Leeuwen et al. 2010). Their findings further confirmed the extreme divergence across PBV genomes, with up to 35% amino acid variation in the RdRp gene alone (Leeuwen et al. 2010). However, PCR and cloning-based approaches cannot quantify viral load due to multiple amplification steps and are limited to detecting sequences, not active infection.

More recently, cloning has been used to explore the functional nature of PBV ORFs. A study by Gan and Wang (2023) identified PBV genes encoding proteins homologous to bacteriolytic enzymes using bioinformatics. To validate these findings experimentally, multiple PBV genome sequences were cloned and expressed in E.coli, resulting in cell lysis detectable by OD600 measurements and microscopy (Gan and Wang 2023). This provided the first direct evidence that PBVs may encode lysins, similar to those found in bacteriophages, strongly supporting the hypothesis that PBVs are prokaryotic viruses. Given that lysins can be species-specific (Young 1992), this technique may also offer a targeted strategy for identifying specific bacterial hosts for PBVs in environmental samples.

## Conclusion and future directions

PBVs present a unique and persistent challenge for virologists. Their unculturable nature, highly diverse segmented genome, use of alternative genetic codes, and lack of a clearly defined host have all complicated their detection and classification.

While PCR remains a sensitive and accessible method for PBV detection, it is constrained by genetic variability and dependence on conserved regions. Metagenomic next-generation sequencing has expanded our understanding of PBV diversity, yet it faces obstacles in RNA extraction, host contamination, and database limitations, especially for segmented and highly divergent RNA viruses like PBVs.

Cloning approaches have provided a contemporary avenue for both increasing genomic yield and probing the functional characteristics of PBV genes. Experimental evidence of bacteriolytic activity and expression in *E. coli* strengthens the hypothesis that PBVs may infect prokaryotes, although further research is required to confirm this. Key questions that remain unresolved:

1. What is the definitive host of PBVs – eukaryotic, prokaryotic, or both?
2. Do PBVs represent the first known virus family to cross domain boundaries?
3. How extensive is the genomic variation within and across PBV genogroups?
4. Are PBVs true pathogens, opportunistic agents, or harmless symbionts?

While these questions require a great amount of additional research, an integrated approach will be essential, combining traditional techniques (PCR, culturing), modern molecular tools (metagenomics) and emerging methods (functional genomics, codon usage bias, and host-linked omics). An example of future techniques that could be used includes single-cell metagenomics and CRISPR-spacer mapping to co-localize PBV genomes with potential hosts. The ongoing study of PBVs offers not only an opportunity to uncover a potentially novel class of RNA viruses but also to push the boundaries of how we detect, classify and understand the viral world.

## Supplementary Material

Supp 1

Supplemental data for this article can be accessed online at https://doi.org/10.1080/1040841X.2025.2560918.

## Data Availability

All sequence data used in this review was obtained from previously published sources (Sadiq et al. 2024) and are publicly available in GenBank. Accession numbers are provided in Supplementary Table 1.
